# Supplementary material for: Modeling glioblastoma heterogeneity as a dynamic network of cell states
Source: Mol Syst Biol. 2021 Sep 16;17(9):e10105. doi: 10.15252/msb.202010105 (PMC8444284; doi:10.15252/msb.202010105)
Supplement: Supplementary file 6 — Source Data for Figure 5 [file MSB-17-e10105-s004.zip › Figure5A_sourcedata/GSEA_3071/nosigdb_stateVI.GseaPreranked.1622795169056/NB_KI_CLUSTER13.html]

Details for gene set NB\_KI\_CLUSTER13[GSEA]

|  || Dataset | state63071 |
| Phenotype | NoPhenotypeAvailable |
| Upregulated in class | na\_pos |
| GeneSet | NB\_KI\_CLUSTER13 |
| Enrichment Score (ES) | 0.23186167 |
| Normalized Enrichment Score (NES) | 1.5590029 |
| Nominal p-value | 0.018292682 |
| FDR q-value | 0.055666156 |
| FWER p-Value | 0.531 |
Table: GSEA Results Summary

  

Fig 1: Enrichment plot: NB\_KI\_CLUSTER13      
 Profile of the Running ES Score & Positions of GeneSet Members on the Rank Ordered List

  

| PROBE | GENE SYMBOL | GENE\_TITLE | RANK IN GENE LIST | RANK METRIC SCORE | RUNNING ES | CORE ENRICHMENT || 1 | CAVIN2 |  |  | 11 | 1.116 | 0.0302 | Yes |
| 2 | HOPX |  |  | 89 | 0.761 | 0.0314 | Yes |
| 3 | DUSP1 |  |  | 136 | 0.685 | 0.0389 | Yes |
| 4 | SQSTM1 |  |  | 147 | 0.667 | 0.0561 | Yes |
| 5 | ELL2 |  |  | 156 | 0.655 | 0.0734 | Yes |
| 6 | DUSP5 |  |  | 169 | 0.631 | 0.0888 | Yes |
| 7 | HBEGF |  |  | 172 | 0.627 | 0.1070 | Yes |
| 8 | SERPINB1 |  |  | 184 | 0.618 | 0.1223 | Yes |
| 9 | NABP1 |  |  | 186 | 0.609 | 0.1402 | Yes |
| 10 | SMOX |  |  | 196 | 0.604 | 0.1558 | Yes |
| 11 | HOTAIRM1 |  |  | 241 | 0.571 | 0.1605 | Yes |
| 12 | MAFF |  |  | 253 | 0.562 | 0.1742 | Yes |
| 13 | AC016831.1 |  |  | 266 | 0.556 | 0.1874 | Yes |
| 14 | USP53 |  |  | 340 | 0.515 | 0.1823 | Yes |
| 15 | CNN2 |  |  | 349 | 0.512 | 0.1954 | Yes |
| 16 | XBP1 |  |  | 389 | 0.494 | 0.1992 | Yes |
| 17 | NR3C1 |  |  | 395 | 0.492 | 0.2124 | Yes |
| 18 | TXNIP |  |  | 402 | 0.487 | 0.2253 | Yes |
| 19 | SOCS1 |  |  | 489 | 0.458 | 0.2149 | Yes |
| 20 | CMTM7 |  |  | 546 | 0.442 | 0.2124 | Yes |
| 21 | TNFSF10 |  |  | 631 | 0.416 | 0.2013 | Yes |
| 22 | AC015912.3 |  |  | 656 | 0.411 | 0.2068 | Yes |
| 23 | GBP3 |  |  | 755 | 0.385 | 0.1909 | Yes |
| 24 | METRNL |  |  | 766 | 0.383 | 0.1995 | Yes |
| 25 | RAP1A |  |  | 782 | 0.380 | 0.2066 | Yes |
| 26 | RAP2B |  |  | 802 | 0.376 | 0.2125 | Yes |
| 27 | HERPUD1 |  |  | 830 | 0.371 | 0.2160 | Yes |
| 28 | CAPG |  |  | 851 | 0.368 | 0.2214 | Yes |
| 29 | KLF6 |  |  | 854 | 0.368 | 0.2319 | Yes |
| 30 | GMDS |  |  | 900 | 0.361 | 0.2300 | No |
| 31 | EPSTI1 |  |  | 955 | 0.352 | 0.2254 | No |
| 32 | TRAF1 |  |  | 1040 | 0.339 | 0.2120 | No |
| 33 | LSP1 |  |  | 1048 | 0.337 | 0.2201 | No |
| 34 | CAST |  |  | 1070 | 0.334 | 0.2242 | No |
| 35 | GYG1 |  |  | 1233 | 0.311 | 0.1881 | No |
| 36 | S1PR1 |  |  | 1306 | 0.302 | 0.1769 | No |
| 37 | ITPR3 |  |  | 1338 | 0.300 | 0.1772 | No |
| 38 | AC016831.5 |  |  | 1339 | 0.299 | 0.1861 | No |
| 39 | GLIPR2 |  |  | 1377 | 0.294 | 0.1845 | No |
| 40 | SERP1 |  |  | 1428 | 0.288 | 0.1791 | No |
| 41 | RAP1B |  |  | 1467 | 0.283 | 0.1769 | No |
| 42 | MAN2A1 |  |  | 1507 | 0.279 | 0.1743 | No |
| 43 | HPS1 |  |  | 1549 | 0.274 | 0.1710 | No |
| 44 | KRT18 |  |  | 1570 | 0.272 | 0.1735 | No |
| 45 | CLCF1 |  |  | 1592 | 0.269 | 0.1757 | No |
| 46 | MGAT1 |  |  | 1649 | 0.264 | 0.1679 | No |
| 47 | ANKRD28 |  |  | 1666 | 0.263 | 0.1712 | No |
| 48 | SLC10A3 |  |  | 1708 | 0.260 | 0.1675 | No |
| 49 | IFI44L |  |  | 1742 | 0.256 | 0.1659 | No |
| 50 | TAPBP |  |  | 1817 | -0.252 | 0.1527 | No |
| 51 | PARP14 |  |  | 2145 | -0.304 | 0.0702 | No |
| 52 | MIR181A1HG |  |  | 2238 | -0.321 | 0.0540 | No |
| 53 | RNF213 |  |  | 2383 | -0.350 | 0.0241 | No |
| 54 | RSRP1 |  |  | 2611 | -0.403 | -0.0275 | No |
| 55 | MAF |  |  | 2633 | -0.408 | -0.0212 | No |
| 56 | SELENOK |  |  | 2831 | -0.467 | -0.0624 | No |
| 57 | CALCRL |  |  | 2853 | -0.478 | -0.0541 | No |
| 58 | CFLAR |  |  | 2902 | -0.495 | -0.0527 | No |
| 59 | DNASE2 |  |  | 2924 | -0.503 | -0.0436 | No |
| 60 | ARHGEF6 |  |  | 3003 | -0.544 | -0.0492 | No |
| 61 | ID2 |  |  | 3026 | -0.554 | -0.0389 | No |
| 62 | LEF1 |  |  | 3149 | -0.616 | -0.0546 | No |
| 63 | PTPRJ |  |  | 3174 | -0.640 | -0.0423 | No |
| 64 | SKIL |  |  | 3188 | -0.652 | -0.0265 | No |
| 65 | JUND |  |  | 3295 | -0.755 | -0.0336 | No |
| 66 | BTG2 |  |  | 3377 | -0.857 | -0.0307 | No |
| 67 | CD82 |  |  | 3408 | -0.912 | -0.0119 | No |
| 68 | DUSP6 |  |  | 3512 | -1.151 | -0.0064 | No |
| 69 | ITPR2 |  |  | 3556 | -1.388 | 0.0230 | No |
Table: GSEA details [plain text format]

  

Fig 2: NB\_KI\_CLUSTER13: Random ES distribution      
 Gene set null distribution of ES for **NB\_KI\_CLUSTER13**
